# Etiology of ‘Sinus Headache’—Moving the Focus from Rhinology to Neurology. A Systematic Review

**DOI:** 10.3390/brainsci11010079

**Published:** 2021-01-09

**Authors:** Marcin Straburzyński, Anna Gryglas-Dworak, Magdalena Nowaczewska, Eliza Brożek-Mądry, Paolo Martelletti

**Affiliations:** 1 Headache Clinic—Terapia Neurologiczna Samodzielni, 02-042 Warsaw, Poland; 2 Department of Pediatrics and Rare Diseases, Headache Center Wroclaw, Wroclaw Medical University, 51-618 Wroclaw, Poland; anna.gryglas@gmail.com; 3 Department of Otolaryngology, Head and Neck Surgery and Laryngological Oncology, Ludwik Rydygier Collegium Medicum in Bydgoszcz, Nicolaus Copernicus University, 85-094 Bydgoszcz, Poland; magy_mat@by.onet.pl; 4 Department of Otorhinolaryngology, Faculty of Medicine and Dentistry, Medical University of Warsaw, 00-739 Warsaw, Poland; eliza.madry@gmail.com; 5 Department of Clinical and Molecular Medicine, Sapienza University, 00189 Rome, Italy; paolo.martelletti@uniroma1.it; 6 Emergency Medicine Unit, Regional Referral Headache Centre, DAI Medical Sciences, Sant’Andrea Hospital, 00189 Rome, Italy

**Keywords:** sinus headache, migraine, tension-type headache, sinusitis, facial pain, midfacial segment pain

## Abstract

‘Sinus headache and/or facial pain’ (SH) is a common complaint encountered by otorhinolaryngologists, neurologists and general practitioners. However, several studies suggested that the majority of those cases may be attributed to primary headaches (i.e., migraine and tension-type headache (TTH). The purpose of this review is to evaluate the etiology of SH. The first part includes cross-sectional studies analyzing the prevalence of respective diagnoses in subjects with SH. The majority of these publications indicate that migraine and TTH are the most prevalent causes of SH, although most of these studies were conducted in a clinical setting. The second part of this review included treatment trials in subjects with SH. The findings from this part of the review show that SH without rhinosinusitis responds well to pharmacotherapy targeted at primary headaches. This observation further supports a neurologic etiology of the majority of SH cases.

## 1. Introduction

Sinonasal etiology of headache and facial pain is often suspected in otorhinolaryngologic, neurologic or general practice. However, establishing a diagnosis is sometimes challenging even for experienced physicians. Misunderstandings often start with patients receiving an incorrect diagnosis or trying to diagnose themselves. In these instances, ‘sinus headache’ (SH) and/or ‘sinus facial pain’ is suspected. The rationale behind this assumption lies in clinical similarities between rhinogenic and neurogenic headaches: both may cause pain located directly over paranasal sinuses and both can be accompanied by nasal discharge and congestion [1]. That is why in many cases SH eventually turns out to be a neurologic disorder misattributed to rhinologic diseases [2], and patients often wait decades for adequate treatment [3]. These observations are supported by the results of a population-based study, where 36.5% of patients fulfilling criteria of migraine had been previously diagnosed with SH [4]. That proportion was even higher (42%) in a similar study conducted almost two decades earlier [5]. Those results were confirmed by clinical observations: in a retrospective analysis of 130 migraine patients, 81.5% had been previously diagnosed with SH [6]. Consequently, according to some authors, SH is the most common misdiagnosis in migraine [4]. Several narrative reviews and two evidence-based reviews addressed this subject in the past. However, they used different inclusion criteria and consequently analyzed fewer studies [7] or only pediatric population [8]. To establish the prevalence of particular diagnoses in patients complaining of SH, the authors reviewed cross-sectional studies in different clinical settings or among the general population. Furthermore, the second part of this review includes treatment trials evaluating the effects of pharmacotherapy targeted at primary headaches in subjects with SH.

## 2. Materials and Methods

This systematic review followed the Reporting Items for Systematic reviews and Meta-Analyses (PRISMA) statement [9]. Cross-sectional studies that establish etiology of complaints in patients reporting with SH were eligible for this evaluation. The second part of this paper includes case series, case control studies and clinical trials evaluating the effects of pharmacotherapy in subjects with SH. This review only includes articles published in English. The authors did not make any additional restrictions as to the publication date, patients’ gender, age, recruitment methods or type of intervention (for clinical trials). However, case reports and studies with too little data for analyzing SH etiology were excluded.

The authors eliminated studies in which headache or facial pain was not the principal complaint, but a part of a clinical picture indicating a true rhinologic disease. This was done to better translate the results from this review to clinical situations in which patients report to their doctors with SH as their primary complaint. However, a debatable issue stands between a clear case of sinonasal disease (i.e., rhinosinusitis) and healthy nasal cavity and sinuses: mucosal contact points (MCP). These are anatomical variations of the nasal cavity, resulting in contact between two anatomical structures that do not adhere in most patients (e.g., nasal turbinates connecting with nasal septum). The dispute regarding whether MCP can be a source of pain is still ongoing, with a large body of mostly low grade evidence pointing to the effectiveness of surgery [10]. However, guidelines discourage surgical treatment in these subjects, pointing out that the evidence is of low quality and pain remission may be attributed to a placebo effect, neuromodulation or other factors unrelated directly to the removal of MCP [11,12]. To avoid this moot point, we have excluded MCP surgery and local anesthesia studies from our review.

The following databases were screened: PubMed, Google Scholar and the Cochrane database library. The MS and MN performed independently an unblinded eligibility assessment of obtained records. The last search was performed on 10/02/2020. The articles were searched for the following terms: ‘sinus’ OR ‘paranasal sinuses’ OR ‘nose’ OR ‘nasal’ AND ‘headache’ OR ‘pain’. Titles and abstracts in records obtained from databases were evaluated to select only studies meeting the inclusion criteria. After that, the authors read and screened the full texts of records that were not rejected in the previous stage to assess if they fulfilled inclusion or exclusion criteria. Reference lists of evaluated full-text articles and relevant reviews were screened for potentially omitted studies. When multiple publications described the same study group, only the one with most exhaustive data was chosen for this review, unless the perspectives from different publications provided new information.

The following information was extracted from each cross-sectional study: recruitment method, inclusion and exclusion criteria, population demographics (percentage of female participants, age), prior treatments, final diagnosis and methods used for establishing it. The latter point encompassed especially whether otorhinolaryngologic evaluation (ENT) or neurologic consultation was obtained, and diagnostic procedures performed in all subjects (i.e., nasal endoscopic examination, paranasal sinus computed tomography (CT) or magnetic resonance imaging). In regard to clinical trials, the authors also extracted the type of intervention applied and its results. Risk of bias for prevalence studies was assessed according to the modified method described by Hoy et al. [13] (see: Supplementary Materials File 1). In case of randomized clinical trials, The Cochrane Collaboration’s tool for assessing risk of bias was used [14].

Due to highly inclusive criteria, the authors expected heterogenous data in respect to patients’ assessment and treatment, therefore a meta-analysis could not be considered. As a consequence, the primary outcome of this review would be a descriptive analysis pointing out the major causes of SH. These observations should be supported by effectiveness in SH of treatment targeted at primary headaches. To our knowledge it is the first review addressing this subject in this systematic manner. 

## 3. Results

### 3.1. Causes of Complaints in Patients with ‘Sinus Headache’

**Figure 1:**
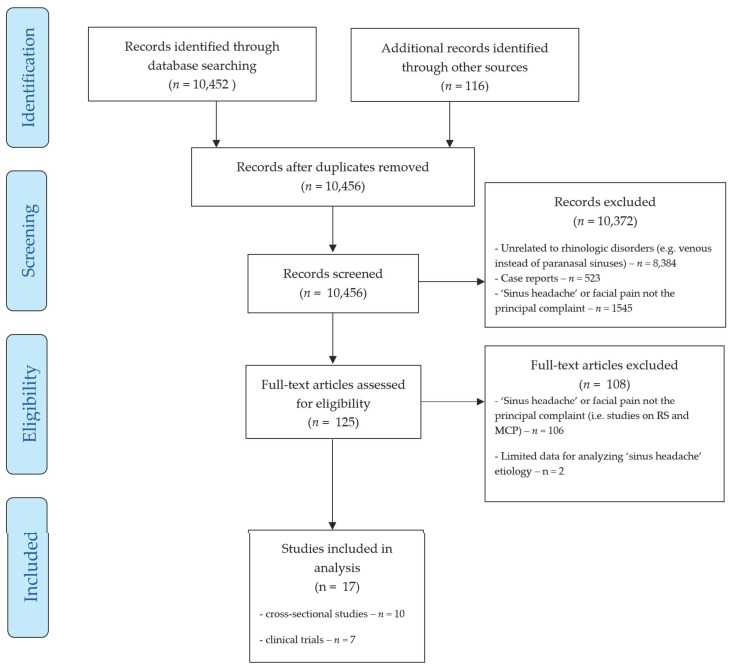
Study selection diagram. RS—rhinosinusitis, MCP—mucosal contact point. (see Supplementary Materials File 2 for the list of full-text articles excluded).

Studies included in this part of the review present data on patients reporting for a headache-related medical evaluation. Figure 1 presents the flow diagram describing selection of studies according to PRISMA statement. Patients either described their complaints as SH, or were referred with suspected rhinogenic headache and/or facial pain by another physician. Studies that were included in this part of the review are listed in Table 1. According to adopted review protocol, these do not include case series of patients without suspected rhinogenic headache as their primary complaint [6], or cases where the inclusion criteria were too restrictive for the analysis of SH etiology [15]. 

This review highlights the fact that the majority of studies recognize migraine as the cause of complaints in patients reporting with ‘sinus headache’. This observation was valid even for studies in which prior diagnosis of migraine was one of the exclusion criteria [16,17] (Table 1). In other words, previously unrecognized migraine is highly prevalent among patients reporting with ‘sinus headache’.

Studies described in this review applied different inclusion and exclusion criteria. To better understand what causes SH, some studies recruited only subjects without rhinologic symptoms or radiographic signs of rhinosinustits [16,17]. Other studies had wider inclusion criteria, but allowed for the analysis of data for subjects without rhinologic disease [3,18,19,22]. This course of action confirmed that the majority of SH are not caused by sinonasal disorders. However, excluding subjects with signs of rhinosinusitis might have led to an underestimation of primary headache prevalence in SH cohort. This could be a result of incidental rhinologic signs and symptoms accompanying primary headache disorders. After all, it must be remembered that nasal congestion or discharge, as well as CT changes, may occur in patients without sinonasal disorders [25,26,27]. This concept is supported by results from one of the larger groups included in this review [18,28]. In this study facial pain or headache was unrelated to rhinosinusitis in 75% of the subgroup with abnormal results of endoscopic examination. This conclusion was drawn as these subjects did not respond to either medical or surgical rhinologic treatment.

‘Sinus headache’ patients were recruited from ENT or neurologic clinics with one exception that involved volunteers from the general population [3]. Hence only the latter study (Sinus, Allergy and Migraine Study—SAMS) avoided bias related to the selection of patients from a clinical setting. Neither previous diagnosis of migraine nor evidence of rhinosinusitis excluded participants from SAMS. The majority of studies recruited adults, and only one of them analyzed exclusively children and adolescents [22]. However, the results from the latter study were consistent with studies in adults [3,16,17]. 

Studies included in this review present different approaches to the diagnostic evaluation of patients (Table 1). In some of them, neurologic or ENT consultation was optional or absent. Headache specialists were involved in just three of the included studies [3,17,24]. Moreover, nasal endoscopy or CT were not universally performed. As a consequence, most authors were unable to avoid bias resulting from gaps in clinical data. For example, detailed analysis shows that primary headaches might have been responsible for complaints of a higher number of patients in some studies, especially considering possible cranial autonomic symptoms (CAS) that occur in migraine [19,20]. In one series of patients [18,28] subjects with normal results from endoscopic examination and CT were diagnosed with migraine in 10% and TTH or midfacial pain in 51%. The reason for such a small prevalence of migraine is unclear, although the lack of headache specialist evaluation might be significant. On the other hand, even the inclusion of general neurologic consultation did not secure full adherence to the International Classification of Headache Disorders in establishing diagnosis [19]. Since SH is a symptom subject to both headache therapy and rhinology, expertise from two areas is needed for a correct diagnosis. This rationale implicates also that rhinologist input is important, as neurologists may underestimate the value of modern rhinologic evaluation, i.e., nasal endoscopy or CT instead of the now obsolete plain sinus X-rays [6]. In conclusion, it seems that lack of a multidisciplinary approach might have resulted in limited diagnostic accuracy in some of studies included in the review (Table 1).

Facial location and accompanying CAS are the only features of SH that could be confirmed after the analysis of data provided in studies included in this review. Other pain characteristics, if available, show few similarities. For example in SAMS [3], patients with migraine had on average 14 headache days per month, a value much higher than in the Foroughipour et al. study [16]. There is no data on the prevalence of chronic migraine in studies included in this review, although there are reports that chronic migraine is significantly more prevalent in migraine patients previously diagnosed with SH [6].

One of the studies included in this part of the review introduced a diagnosis of midfacial pain (MFP) and showed high prevalence of this disorder in patients with suspected rhinogenic facial pain [18]. MFP was defined as symmetric facial pain or pressure with normal results of CT and nasal endoscopic examination, but without accompanying nasal symptoms. It should be virtually identical to TTH, but localized in the face, so according to recently published classification it can be attributed to tension-type orofacial pain [29]. Another similarity between TTH and MFP is facial hypersensitivity to touch in MFP, which could be an equivalent of myofascial tenderness in TTH. On a side note it should be mentioned that MFP is a term still used by some authors. One study not included in this review provides interesting insight in MFP characteristics. This was a ENT clinic-based study that included 240 subjects with chronic facial pain [15]. This study was not included in Table 1, as it had restrictive inclusion criteria in respect to pain characteristics. Moreover, although the authors excluded subjects with temporo-mandibular disorders or rhinosinusitis, rhinitis was not among exclusion criteria. The patients were diagnosed with chronic MFP (65%), facial migraine (26%), co-existing MFP and migraine (3%), as well as other pain-related disorders (6%). In other words. the results from that study correspond with one of the studies included in this review [18]. Interestingly, dizziness was an accompanying symptom in 24% MFP subjects, and some patients reported facial oedema [15].

Some of the studies included in this review provide data on previous treatments received by patients. One study noted that 22% percent of SH patients had prior nasal surgery, however, pain was the principal indication for those procedures in merely 6% of subjects [3]. In another study, 16% of patients had undergone septoplasty in the past [16]. Moreover, 96% of ‘sinus headache’ patients were at least once unnecessarily treated with antibiotics [16], an observation valid also for children [22]. The most recent of these studies showed that 41% SH subjects had previously undergone sinonasal surgery (endoscopic in 26% cases) [24].

Data on trigeminal autonomic cephalalgias (TACs) as an underlying problem in ‘sinus headache’ patients is limited. Our review shows that TACs can be responsible for up to 11% of SH cases. Interestingly, studies based in ENT clinics tend to report a higher prevalence of TACs (Table 1). It seems that further evaluation of those subjects by headache specialists could provide valuable data, especially since neurologists report that in 21–23% of cases cluster headache is initially mistaken for sinusitis [30,31,32], an error that may even be found in recently published reports [33]. Moreover, 3–12% patients fulfilling cluster headache criteria undergo unnecessary sinonasal surgery [30,31,34,35]. Similarly, in a study of hemicrania continua, 20% of patients were at first misdiagnosed with SH and 16% underwent sinonasal surgery [36].

The burden of SH was measured by two ENT-based studies [20,24]. Both of them used the Sino-Nasal Outcome Test (SNOT-20 and SONT-22), a tool validated for assessing symptoms in subjects with rhinologic disorders. Patients eventually diagnosed with migraine or TTH had significantly higher scores in this test, especially in domains dedicated to reduced quality of life. Additionally, anxiety (65%) and depression (39%) had high prevalence in one study of SH subjects [24]. On a side note it should be mentioned that SNOT shows that compared to chronic rhinosinusitis (CRS), MFP patients experience a heavier psychologic rather than rhinologic burden [37]. This observation proves another similarity between MFP and primary headache disorders.

### 3.2. Treatment Approaches to ‘Sinus Headache’

This part of the review focuses on studies evaluating the treatment of patients with self-described SH with medications effective in primary headache disorders (Table 2). As shown in the first part of this review, SH is not a diagnosis, but rather a set of symptoms mostly misattributed to sinonasal disorders. Hence the management of this condition requires addressing the underlying disorder. Bearing that in mind, some authors have published management trials targeted at the neurologic etiology of SH. Theoretically this can support the accuracy of diagnosis. This way of thinking applies ex juvantibus diagnosis, i.e., establishing the cause of disease on the basis of the effects of treatment. However, it requires disease-specific therapy to be accurate. In other words, migraine medications should be ineffective in sinonasal diseases. Meanwhile, there is limited data on the effects of migraine therapies (i.e., triptans) in rhinosinusitis. A positive effect could however be expected, considering that triptans are serotonin-receptor agonists, and as such may alleviate pain resulting from trigeminal complex activation [26]. In rhinosinusitis such activation is present, therefore triptans could be effective. That is one of the reasons why studies included in this part of the review almost unanimously excluded subjects with evidence of rhinologic disease.

Several studies evaluated triptans in SH [1,38,39,40] (Table 2). Those included two case series, one case-control study and one randomized control trial. All of the studies show some risk of bias in respect to patient selection (retrospective inclusion) [41], patient definition (limited data on patients demographics or gaps in rhinologic evaluation) [1,40] and end-point choice (degree of pain decrease and time-to-end-point) [1,39,40,41]. The most important of those studies was a randomized controlled trial [38]. In this group, 215 SH subjects with normal CT and no discolored discharge were randomized into a sumatriptan (50 mg) or placebo group. CAS prevalence was high (66 to 79%) similarly to the Cady and Schreiber group. Reduction of pain, but not CAS, was significantly greater in patients receiving triptans. The risk of bias of this trial was assessed as follows: random sequence generation: low, allocation concealment: low, blinding of participants and personnel: low, blinding of outcome assessment: unclear, incomplete outcome data: low, selective reporting: low, other bias: low.

Interestingly, several authors have also published data on migraine abortive medications in subjects with rhinosinusitis. One such case series describes 52 migraine patients who did not respond to surgical or medical rhinosinusitis treatment, despite the fact that 29% had concomitant rhinologic diseases (i.e., allergic rhinitis, CRS with polyps). Finally the patients were prescribed triptans or ergotamine with improvement observed in all cases [28]. Furthermore an interesting approach was presented in another study [41]. This research included patients with migraine with co-existing CRS (*n* = 29) or without CRS (*n* = 45). Patients from both groups received eletriptan as an abortive treatment. Response to this drug was similar in both groups, which suggests that pain in CRS was unrelated to sinonasal disease or that triptans can also improve rhinosinusitis-related headache.

Migraine preventive medications were tested in patients with SH by one study [42]. In this prospective evaluation 72 subjects received 500 mg of sodium valproate per day for three months. 86.1% of subjects completed the study and 61.1% observed at least 50% reduction of pain severity and frequency.

One of the principles of this review is the exclusion of MCP surgery studies [43,44,45,46,47,48,49,50,51,52,53,54,55,56,57,58]. There are several reasons for that approach. Firstly, none of the cross-sectional studies identified in this literature search indicated MCP as a source of headache (see Section 3.1 of this review). Since most of these studies were performed in ENT clinics, it indicates that many surgeons do not consider MCP a cause of SH. This is understandable, as there are no population-based studies that show that MCP increases the risk of headache. Additionally, there is no ‘MCP headache’ listed in the current International Classification of Headache Disorders; headache attributed to MCP was described in the appendix as pending confirmation of its existence [59]. Consequently, the recent European guidelines do not recommend surgery for the treatment of headache or facial pain [11,12]. On the other hand, there exists a large group of authors who are proponents of MCP headache surgery [43,44,45,46,47,48,50,51,52,53,54,55,56,57,58]. These authors indicate excellent results of case series and low-grade controlled trials. This evidence should not be disregarded, as it suggests that MCP is a candidate for etiological factor of SH. However, to confirm this, a cause and effect relationship should be established. That can be achieved by high quality scientific evidence. The importance of good-quality evidence in SH surgery can be exemplified by the only single-blinded randomized controlled trial published so far. In this study, balloon sinuplasty had no advantage over sham intervention in reducing sinus pressure or headache in otherwise rhinologically-healthy subjects [49].

## 4. Conclusions

- Migraine and tension-type headache are the most prevalent causes of complaints in patients reporting to a physician for treatment of ‘sinus headache and facial pain’.
- Medications effective in migraine and tension-type headache also decrease ‘sinus headache’ symptoms. Although this finding cannot be treated as diagnosis confirmation, it supports observations that ‘sinus headache’ is mostly misdiagnosed primary headache.
- Multidisciplinary evaluation of patients with ‘sinus headache’ should be introduced at an early stage. The team should include a headache specialist and rhinologist, as this would allow for the correct classification of headache type and avoidance of unnecessary surgical procedures.

## Figures and Tables

**Table 1:** Studies evaluating etiology of ‘sinus headache’. TTH—tension-type headache, TACs—trigeminal autonomic cephalalgias, n/a—data not available, N—neurologic evaluation, ENT—otorhinolaryngologic evaluation, CT—computed tomography of paranasal sinuses, NE—nasal endoscopy, * including midfacial pain.

	Patient Selection	Evaluation of the Entire Study Group	Rhinosinusitis Excluded?	Female/Male (%)	Age	No. of Participants	Migraine (incl. Probable Migraine) (%)	TTH (%)	TACs (%)	Rhinogenic (%)	Other or Unspecified (%)	Bias Risk
[18]	ENT clinic	N − ENT + CT+, NE+	No	69/31	16–56	409	12	42 *	6	19	21	Moderate
[19]	ENT clinic	N + ENT + CT +, NE+	Yes	78/22	22–85	75	37	12	-	-	51	Moderate
[20]	ENT clinic	N − ENT + CT +, NE+	n/a	n/a	n/a	69	30	3	1	48	18	High
[17]	Headache clinic	N + ENT − CT −, NE−	Yes	77/23	18–65	2991	88	8	-	-	4	Moderate
[3]	General population	N + ENT − CT −, NE−	No	78/22	18–81	100	86	-	2	3	9	Low
[21]	ENT clinic	N − ENT + CT +, NE−	No	n/a	n/a	35	74	n/a	n/a	n/a	26	Moderate
[22]	N clinic	N + ENT − CT −, NE−	No	54/46	4–16	87	77	19	-	2	2	Moderate
[16]	ENT clinic	N + ENT + CT +, NE−	Yes	62/38	10–65	58	68	27	-	5	-	Moderate
[23]	ENT clinic	N − ENT+ CT +, NE+	Yes	72/28	17–63	103	62	25	11	-	3	Moderate
[24]	ENT clinic	N + ENT + CT +, NE+	Yes	67/33	n/a	46	65	15	-	-	20	Moderate

**Table 2:** Abortive migraine treatment trials in ‘sinus headache’. p.o.—per os, n/a—data not available. CRS—chronic rhinosinusitis.

	Type of Intervention	Study Design	Group Selection	No. of Participants (with Intention to Treat)	Rhinosinusitis Patients Excluded?	Female/Male (%)	Age	Outcome
[1]	Sumatriptan 50 mg p.o.	Case series	General population	37 (47)	Yes	n/a	n/a	Significant reduction of headache in 100% of moderate/severe attacks
[39]	Eletriptan 40 mg p.o.	Case series	ENT clinic	38 (55)	Yes	67/33	23–70 (median 39)	Significant reduction of headache in 81.6% of attacks
[38]	Sumatriptan 50 mg p.o.	Randomized double-blind placebo controlled	n/a	213 (215)	Yes	70/30	18–70 (median 42)	Significant reduction of headache in 69% vs. 47% (*p* < 0.001) of patients
[41]	Eletriptan p.o. (dose n/a)	Retrospective case-control study	ENT clinic	29 (67)	No	55/45	18–81 (mean 49)	Resolution of headache in 82.8% of CRS migraine subjects (no significant difference with control group of migraineurs without CRS

## Supplementary Materials

The following are available online at https://www.mdpi.com/2076-3425/11/1/79/s1: Supplementary Materials file 1: Prevalence studies bias risk assessment; Supplementary Materials file 2: List of references of full text studies screened in the selection process.
