# Supplementary material for: Etiology of ‘Sinus Headache’—Moving the Focus from Rhinology to Neurology. A Systematic Review
Source: Brain Sci. 2021 Jan 9;11(1):79. doi: 10.3390/brainsci11010079 (PMC7827425; doi:10.3390/brainsci11010079)
Supplement: Supplementary file 1 [file brainsci-11-00079-s001.zip › brainsci-1048792-supplement 1-grading table.pdf]

brain sciences

Review

Etiology of 'Sinus Headache'—Moving the Focus from Rhinology to Neurology. A Systematic Review

SUPLEMMENTARY MATERIALS

PREVALENCE STUDIES BIAS RISK ASSESSMENT

Straburzyński Marcin, Gryglas-Dworak Anna, Nowaczewska Magdalena, Brożek-Mądry Eliza and Martelletti Paolo

Table 1. Assessment of the risk of bias in prevalence studies of 'sinus headache'.

Item (column):

A. Was the sampling frame a true or close representation of the target population (i.e. patients complaining 'sinus headache and/or facial pain' (SH)? - "No" was marked when important data concerning population was lacking.

B. Was some form of random selection used to select the sample, or was a census undertaken?

C. Was the likelihood of non-response bias minimal? - "No" was marked in all studies that did not provide data on number of subjects lost to follow-up during diagnostic process or if number lost to follow-up was higher than 20%.

D. Was data collected directly from the participants (as opposed to a proxy)?

E. Was the study's target population a close representation of the analyzed population in relation to relevant variables? - "No" was marked if authors presented no data regarding this subject or no comparable control group was present.

F. Was an acceptable case definition used in the study? – "Yes" was marked if diagnosis was consistent with recommended disease definitions (i.e. International Classification of Headache Disorders, sinusitis confirmed/excluded by either nasal endoscopy or/and computed tomography (CT)).

G. Was the study instrument that measured the parameter of interest shown to have validity and reliability? – "Yes" was marked if the diagnosis was established by a physician with strict adherence to disease definitions.

H. Was the same mode of data collection used for all participants? – "No" was marked if any diagnostic procedures were omitted in some of the subjects.

I. Was the length of the shortest prevalence period for the parameter of interest appropriate?- "Yes" was marked in all cases of appropriately established diagnosis

J. Where the numerator(s) and denominator(s) for the parameter of interest appropriate?

	A	B	C	D	E	F	G	H	I	J	Total
[1]	0	1	1	0	1	1	1	0	0	0	4
[2]	0	1	1	0	1	1	1	0	0	0	5
[3]	1	1	1	0	1	1	1	1	0	0	7
[4]	0	1	1	0	1	1	1	0	0	0	5
[5]	0	0	1	0	0	1	1	0	0	0	3
[6]	1	1	1	0	1	0	1	0	0	0	5
[7]	0	1	1	0	1	0	1	1	0	0	5

[8]	0	1	1	0	1	0	0	1	0	0	4
[9]	0	1	1	0	1	0	0	1	0	0	4
[10]	1	1	1	0	1	0	1	1	0	0	6

Low risk	0-3	Yes = 0
Moderate risk	4-6	No = 1
High risk	7-10	

References

1. West, B.; Jones, N.S. Endoscopy-Negative, Computed Tomography-Negative Facial Pain in a Nasal Clinic: Laryngoscope **2001**, 111, 581–586, doi:10.1097/00005537-200104000-00006.
2. Paulson, E.P.; Graham, S.M. Neurologic Diagnosis and Treatment in Patients with Computed Tomography and Nasal En-doscopy Negative Facial Pain: Laryngoscope **2004**, 114, 1992–1996, doi:10.1097/01.mlg.0000147935.59755.45.
3. Perry, B.F.; Login, I.S.; Kountakis, S.E. Nonrhinologic Headache in a Tertiary Rhinology Practice. Otolaryngol. Head Neck Surg. **2004**, 130, 449–452, doi:10.1016/j.otohns.2004.01.005.
4. Schreiber, C.P.; Hutchinson, S.; Webster, C.J.; Ames, M.; Richardson, M.S.; Powers, C. Prevalence of Migraine in Patients With a History of Self-Reported or Physician-Diagnosed "Sinus" Headache. Arch. Intern. Med. **2004**, 164, 1769, doi:10.1001/archinte.164.16.1769.
5. Eross, E.; Dodick, D.; Eross, M. The Sinus, Allergy and Migraine Study (SAMS): CME. *Headache J. Head Face Pain* **2007**, *47*, 213–224, doi:10.1111/j.1526-4610.2006.00688.x.
6. Mehle, M.E.; Kremer, P.S. Sinus CT Scan Findings in "Sinus Headache" Migraineurs. Headache **2007**, 0, 070516095856009, doi:10.1111/j.1526-4610.2007.00811.x.
7. Şenbil, N.; Gürer, Y.K.Y.; Üner, Ç.; Barut, Y. Sinusitis in Children and Adolescents with Chronic or Recurrent Headache: A Case–Control Study. J. Headache Pain **2008**, 9, 33–36, doi:10.1007/s10194-008-0007-0.
8. Foroughipour, M.; Sharifian, S.M.R.; Shoeibi, A.; Ebdali Barabad, N.; Bakhshaee, M. Causes of Headache in Patients with a Primary Diagnosis of Sinus Headache. Eur. Arch. Oto-Rhino-Laryngol. **2011**, 268, 1593–1596, doi:10.1007/s00405-011-1643-6.
9. Kaymakci, M.; Cikriklar, H.I.; Pay, G. The Aetiology Underlying Sinus Headaches. Journal of International Medical Research **2013**, 41, 218–223, doi:10.1177/0300060513475382.
10. Lal, D.; Rounds, A.B.; Rank, M.A.; Divekar, R. Clinical and 22-Item Sino-Nasal Outcome Test Symptom Patterns in Primary Headache Disorder Patients Presenting to Otolaryngologists with "Sinus" Headaches, Pain or Pressure: SNOT-22 Symptoms in Primary Headache Patients. Int. Forum Allergy Rhinol. 2015, 5, 408–416, doi:10.1002/alr.21502.
