# Supplementary material for: Etiology of ‘Sinus Headache’—Moving the Focus from Rhinology to Neurology. A Systematic Review
Source: Brain Sci. 2021 Jan 9;11(1):79. doi: 10.3390/brainsci11010079 (PMC7827425; doi:10.3390/brainsci11010079)
Supplement: Supplementary file 1 [file brainsci-11-00079-s001.zip › brainsci-1048792-supplement 2-references.pdf]

brain sciences

MDPI

Review

Etiology of 'Sinus Headache'—Moving the Focus from Rhinology to Neurology. A Systematic Review

SUPLEMMENTARY MATERIALS

REFERENCES OF FULL TEXT STUDIES SCREENED IN THE SELECTION PROCESS

Straburzyński Marcin, Gryglas-Dworak Anna, Nowaczewska Magdalena, Brożek-Mądry Eliza and Martelletti Paolo

1. Abu-Bakra M, Jones NS. Prevalence of nasal mucosal contact points in patients with facial pain compared with patients without facial pain. J Laryngol Otol 2001;115. https://doi.org/10.1258/0022215011908685.

2. Abu-Samra M, Gawad OA, Agha M. The outcomes for nasal contact point surgeries in patients with unsatisfactory response to chronic daily headache medications. Eur Arch Otorhinolaryngol 2011;268:1299–304. https://doi.org/10.1007/s00405-011-1590-2.

3. Acquadro MA, Montgomery WW. Treatment of Chronic Paranasal Sinus Pain with Minimal Sinus Disease. Ann Otol Rhinol Laryngol 1996;105:607–14. https://doi.org/10.1177/000348949610500804.

4. Acquadro MA, Salman SD, Joseph MP. Analysis of Pain and Endoscopic Sinus Surgery for Sinusitis. Ann Otol Rhinol Laryngol 1997;106:305–9. https://doi.org/10.1177/000348949710600408.

5. Adnan, Ihsanullah, Khan S, Haq A, Javaid, Shahabi I. Rhinogenic headache and its management. J Med Sci (Peshawar, Print) 2015;23:206–8.

6. Afifi AM, Schwarze ML, Stilp EK, Orne J, Smith JP, Abd-Elsayed AA, et al. "Like a Normal Person Again": A Qualitative Analysis of the Impact of Headache Surgery. Plastic and Reconstructive Surgery 2019;144:956–64. https://doi.org/10.1097/PRS.0000000000006071.

7. Agius AM, Jones NS, Muscat R. Prospective three-year follow up of a cohort study of 240 patients with chronic facial pain. J Laryngol Otol 2014;128:518–26. https://doi.org/10.1017/S0022215114000929.

8. Agius AM. Long-term follow-up of patients with facial pain in chronic rhinosinusitis--correlation with nasal endoscopy and CT. Rhinology 2010;48:65–70. https://doi.org/10.4193/Rhin09.015.

9. Al-Hashel JY, Ahmed SF, Alroughani R, Goadsby PJ. Migraine misdiagnosis as a sinusitis, a delay that can last for many years. The Journal of Headache and Pain 2013;14:97. https://doi.org/10.1186/1129-2377-14-97.

10. Al-Obiedi SHI. Sinusitis in self-described and physician diagnosed patients as sinus headache. Tikrit Medical Journal 2012;18:106–14.

11. Albirmawy. Middle Turbinate Evacuation Conchoplasty in Management of Contact-Point Rhinogenic Headache in Children. Int J Clin Pediatr 2012. https://doi.org/10.4021/ijcp53w.

12. Aljeraisi T. Diagnostic Dilemma of Sinus Headache and Migraine. Ulutas Med J 2020;6:107. https://doi.org/10.5455/umj.20200507094603.

13. Anselmo-Lima WT, Oliveira JAA, Speciali JG, Bordini C, Santos AC, Rocha KV, et al. Middle Turbinate Headache Syndrome. Headache 1997;37:102–6. https://doi.org/10.1046/j.1526-4610.1997.3702102.x.

14. Barinsky GL, Hanba C, Svider PF. Rhinogenic Headache in Children and Adolescents. Curr Pain Headache Rep 2020;24:7. https://doi.org/10.1007/s11916-020-0839-0.

15. Behin F, Behin B, Bigal M, Lipton R. Surgical Treatment of Patients with Refractory Migraine Headaches and Intranasal Contact Points. Cephalalgia 2005;25:439–43. https://doi.org/10.1111/j.1468-2982.2004.00877.x.

16. Behin F, Behin B, Behin D, Baredes S. Surgical Management of Contact Point Headaches. Headache: The Journal of Head and Face Pain 2005;45:204–10. https://doi.org/10.1111/j.1526-4610.2005.05045.x.

17. Bektas D, Alioglu Z, Akyol N, Ural A, Bahadir O, Caylan R. Surgical Outcomes for Rhinogenic Contact Point Headaches. Med Princ Pract 2011;20:29–33. https://doi.org/10.1159/000322076.

18. Bilal N, Selcuk A, Karakus MF, Ikinciogullari A, Ensari S, Dere H. Impact of Corrective Rhinologic Surgery on Rhinogenic Headache: Journal of Craniofacial Surgery 2013;24:1688–91. https://doi.org/10.1097/SCS.0b013e31827c7d8f.

19. Boonchoo R. Functional endoscopic sinus surgery in patients with sinugenic headache. J Med Assoc Thai 1997;80:521–6.

20. Bugten V, Nordgård S, Steinsvåg S. Long-term effects of postoperative measures after sinus surgery. Eur Arch Otorhinolaryngol 2008;265:531–7. https://doi.org/10.1007/s00405-007-0501-z.

21. Cady, R.K.; Schreiber, C.P. Sinus headache: a clinical conundrum. Otolaryngol. Clin. North Am. 2004, 37, 267–288, doi:10.1016/S0030-6665(03)00181-6.

22. Cashman EC, Smyth D. Primary headache syndromes and sinus headache: An approach to diagnosis and management. Auris Nasus Larynx 2012;39:257–60. https://doi.org/10.1016/j.anl.2011.05.003.

23. Charleston L, Strabbing R, Cooper W. Is Sinus Disease the Cause of my Headaches? An Update on Sinus Disease and Headache. Curr Pain Headache Rep 2014;18:418. https://doi.org/10.1007/s11916-014-0418-3.

24. Chester AC, Antisdel JL, Sindwani R. Symptom-specific outcomes of endoscopic sinus surgery: A systematic review. Otolaryngol Head Neck Surg 2009;140:633–9. https://doi.org/10.1016/j.otohns.2008.12.048.

25. Choi JW, Kim EH, Ryu IS, Lim HW, Song YJ, Yeo N-K. Headache Characteristics in Rhinologic Patients and the Role of Surgical Treatment. J Rhinol 2015;22:11. https://doi.org/10.18787/jr.2015.22.1.11.

26. Chow JM. Rhinologic Headaches. Otolaryngol Head Neck Surg 1994;111:211–8. https://doi.org/10.1177/01945998941113P109.

27. Clerico DM. Pneumatized Superior Turbinate as a Cause of Referred Migraine Headache. Laryngoscope 1996;106:874–9. https://doi.org/10.1097/00005537-199607000-00017.

28. Clerico DM. Sinus Headaches Reconsidered: Referred Cephalgia of Rhinologic Origin Masquerading as Refractory Primary Headaches. Headache 1995;35:185–92. https://doi.org/10.1111/j.1526-4610.1995.hed3504185.x.

29. Clifton NJ, Jones NS. Prevalence of facial pain in 108 consecutive patients with paranasal mucopurulent discharge at endoscopy. J Laryngol Otol 2007;121:345–8. https://doi.org/10.1017/S0022215106002647.

30. Cook PR, Nishioka GJ, Davis WE, McKinsey JP. Functional Endoscopic Sinus Surgery in Patients with Normal Computed Tomography Scans. Otolaryngol Head Neck Surg 1994;110:505–9. https://doi.org/10.1177/019459989411000606.

31. Cutler J, Bikhazi N, Light J, Truitt T, Schwartz M, the REMODEL Study Investigators, et al. Standalone Balloon Dilation versus Sinus Surgery for Chronic Rhinosinusitis: A Prospective, Multicenter, Randomized, Controlled Trial. Am J Rhinol�Allergy 2013;27:416–22. https://doi.org/10.2500/ajra.2013.27.3970.

32. Dadgarnia MH, Atighechi S, Baradaranfar MH. The response to sodium valproate of patients with sinus headaches with normal endoscopic and CT findings. Eur Arch Otorhinolaryngol 2010;267:375–9. https://doi.org/10.1007/s00405-009-1090-9.

33. Daudia AT, Jones NS. Facial migraine in a rhinological setting. Clinical Otolaryngology and Allied Sciences 2002;27:521–5. https://doi.org/10.1046/j.1365-2273.2002.00628.x.

34. DeConde AS, Mace JC, Smith TL. The impact of comorbid migraine on quality-of-life outcomes after endoscopic sinus surgery: Impact of Comorbid Migraine on Outcomes of Endoscopic Sinus surgery. The Laryngoscope 2014;124:1750–5. https://doi.org/10.1002/lary.24592.

35. El-Silimy O. The place of endonasal endoscopy in the relief of middle turbinate sinonasal headache syndrome. Rhinology 1995;33:244–5.

36. Eross E, Dodick D, Eross M. The Sinus, Allergy and Migraine Study (SAMS).: CME. Headache: The Journal of Head and Face Pain 2007;47:213–24. https://doi.org/10.1111/j.1526-4610.2006.00688.x.

37. Faleck H, Rothner AD, Erenberg G, Cruse RP. Headache and Subacute Sinusitis in Children and Adolescents. Headache 1988;28:96–8. https://doi.org/10.1111/j.1526-4610.1988.hed2802096.x.

38. Farmer RL, Garg RK, Afifi AM. Can Functional Nasal Surgery Treat Chronic Headaches? A Systematic Review: Plastic and Reconstructive Surgery 2018;142:1583–92. https://doi.org/10.1097/PRS.0000000000004989.

39. Foroughipour M, Sharifian SMR, Shoeibi A, Ebdali Barabad N, Bakhshaee M. Causes of headache in patients with a primary diagnosis of sinus headache. European Archives of Oto-Rhino-Laryngology 2011;268:1593–6. https://doi.org/10.1007/s00405-011-1643-6.

40. Gerbe RW, Fry TL, Fischer ND. Headache of Nasal Spur Origin: an Easily Diagnosed and Surgically Correctable Cause of Facial Pain. Headache 1984;24:329–30. https://doi.org/10.1111/j.1526-4610.1984.hed2406329.x.

41. Ghazipour A, Abshirini H, Hekmat Shoar M, Pursalehan S. Sinonasal headaches and post-operative outcomes after septoplasty in patients with nasal septal deviation. Iran J Otorhinolaryngol 2011;23:133–9.

42. Giacomini PG, Alessandrini M, DePadova A. Septoturbinal surgery in contact point headache syndrome: long-term results. Cranio 2003;21:130–5.

43. Hamilton JG, Haynes SN, Gannon L, Safranek RA. Sinus Headache: A Psychophysiological Study. Headache 1980;20:258–60. https://doi.org/10.1111/j.1526-4610.1980.hed2005258.x.

44. Hammad MS, Gomaa MA. Role of some anatomical nasal abnormalities in rhinogenic headache. Egyptian Journal of Ear, Nose, Throat and Allied Sciences 2012;13:31–5. https://doi.org/10.1016/j.ejenta.2012.01.006.

45. Harley DH, Powitzky ES, Duncavage J. Clinical Outcomes for the Surgical Treatment of Sinonasal Headache. Otolaryngol Head Neck Surg 2003;129:217–21. https://doi.org/10.1016/S0194-5998(03)00623-5.

46. Harrison L, Jones NS. Intranasal contact points as a cause of facial pain or headache: a systematic review. Clinical Otolaryngology 2013;38:8–22. https://doi.org/10.1111/coa.12081.

47. Hoover S. The nasal patho-physiology of headaches and migraines. Diagnosis and treatment of the allergy, infection and nasal septal spurs that cause them. Rhinol Suppl 1987;2:1–23.

48. Huang H-H, Lee T-J, Huang C-C, Chang P-H, Huang S-F. Non–sinusitis-related rhinogenous headache: a ten-year experience. American Journal of Otolaryngology 2008;29:326–32. https://doi.org/10.1016/j.amjoto.2007.10.001.

49. Ishkanian G, Blumenthal H, Webster CJ, Richardson MS, Ames M. Efficacy of sumatriptan tablets in migraineurs self-described or physician-diagnosed as having sinus headache: A randomized, double-blind, placebo-controlled study. Clinical Therapeutics 2007;29:99–109. https://doi.org/10.1016/j.clinthera.2007.01.012.

50. Jayawardena ADL, Chandra R. Headaches and Facial Pain in Rhinology. Am J Rhinol�Allergy 2018;32:12–5. https://doi.org/10.2500/ajra.2018.32.4501.

51. Jones NS. Midfacial segment pain: implications for rhinitis and sinusitis. Curr Allergy Asthma Rep 2004;4:187–92. https://doi.org/10.1007/s11882-004-0025-1.

52. Kari E, DelGaudio JM. Treatment of Sinus Headache as Migraine: The Diagnostic Utility of Triptans: The Laryngoscope 2008;118:2235–9. https://doi.org/10.1097/MLG.0b013e318182f81d.

53. Kaur A, Singh A. Clinical study of headache in relation to sinusitis and its management. J Med Life 2013;6:389–94.

54. Kaymakci M, Cikriklar HI, Pay G. The aetiology underlying sinus headaches. Journal of International Medical Research 2013;41:218–23. https://doi.org/10.1177/0300060513475382.

55. Kirsch CFE. Headache Caused by Sinus Disease. Neuroimaging Clinics of North America 2019;29:227–41. https://doi.org/10.1016/j.nic.2019.01.003.

56. Koch-Henriksen N, Gammelgaard N, Hvidegaard T, Stoksted P. Chronic headache: the role of deformity of the nasal septum. BMJ 1984;288:434–5. https://doi.org/10.1136/bmj.288.6415.434.

57. Kunachak S. Middle Turbinate Lateralization: A Simple Treatment for Rhinologic Headache: The Laryngoscope 2002;112:870–2. https://doi.org/10.1097/00005537-200205000-00017.

58. La Mantia I, Grillo C, Andaloro C. Rhinogenic Contact Point Headache: Surgical Treatment Versus Medical Treatment. Journal of Craniofacial Surgery 2017:1. https://doi.org/10.1097/SCS.0000000000004211.

59. Lal D, Rounds AB, Rank MA, Divekar R. Clinical and 22-item Sino-Nasal Outcome Test symptom patterns in primary headache disorder patients presenting to otolaryngologists with "sinus" headaches, pain or pressure: SNOT-22 symptoms in primary headache patients. International Forum of Allergy & Rhinology 2015;5:408–16. https://doi.org/10.1002/alr.21502.

60. Lal D, Rounds A, Dodick DW. Comprehensive management of patients presenting to the otolaryngologist for Sinus pressure, pain, or headache: Sinus Headache Management. The Laryngoscope 2015;125:303–10. https://doi.org/10.1002/lary.24926.

61. Laury AM, Chen PG, McMains KC. Randomized Controlled Trial Examining the Effects of Balloon Catheter Dilation on "Sinus Pressure" / Barometric Headaches. Otolaryngol Head Neck Surg 2018;159:178–84. https://doi.org/10.1177/0194599818772818.

62. Lee J-H, Kim H-J, Hong Y-H, Kim K-S. Underestimation of Rhinogenic Causes in Patients Presenting to the Emergency Department with Acute Headache. Acta Neurol Taiwan 2015;24:37–42.

63. Lee SH, Kim Y, Lim TY. Efficacy of sphenopalatine ganglion block in nasal mucosal headache presenting as facial pain. CRANIO® 2018:1–3. https://doi.org/10.1080/08869634.2018.1475859.

64. Leong SC, Lazarova L, Tsang HK, Banhegyi G. Treatment outcomes of midfacial segment pain: experience from the Liverpool multi-disciplinary team facial pain clinic. Rhin 2015;53:35–40. https://doi.org/10.4193/Rhin14.156.

65. Leong SC, Tsang HK, Wilkie MD, Banhegyi G. Characterisation of patients with endoscopy-negative, computer tomography-negative midfacial segment pain using the sino-nasal outcome test. Rhin 2014;52:78–83. https://doi.org/10.4193/Rhin13.123.

66. Lin S-H, Ho C-Y. Paranasal Sinus Pathologies in Patients Presenting with Headache as the Primary Symptom. Cephalalgia 2006;26:423–7. https://doi.org/10.1111/j.1468-2982.2005.01058.x.

67. Low WK, Willatt DJ. Headaches Associated With Nasal Obstruction Due to Deviated Nasal Septum. Headache 1995;35:404–6. https://doi.org/10.1111/j.1526-4610.1995.hed3507404.x.

68. Madani SA, Hashemi SA, Morshedzadeh SA. Results of Functional Endoscopic Sinus Surgery in Patients with Mucosal Contact Points Suffering From Chronic Daily Headache Non-Responding to Medications. Acta Facultatis Medicae Naissensis 2013;30:159–64. https://doi.org/10.2478/afmnai-2013-0008.

69. Mariotti LJ, Setliff RC, Ghaderi M, Voth S. Patient history and CT findings in predicting surgical outcomes for patients with rhinogenic headache. Ear Nose Throat J 2009;88:926–9.

70. Marzetti A, Mazzone S, Tedaldi M, Topazio D, Passali FM. The Role of Balloon Sinuplasty in the Treatment of Vacuum Rhinogenic Headache. Indian J Otolaryngol Head Neck Surg 2017;69:216–20. https://doi.org/10.1007/s12070-017-1086-5.

71. Marzetti A, Tedaldi M, Passali FM. The role of balloon sinuplasty in the treatment of sinus headache. Otolaryngologia Polska 2014;68:15–9. https://doi.org/10.1016/j.otpol.2013.10.005.

72. Maurya A, Qureshi S, Jadia S, Maurya M. "Sinus Headache": Diagnosis and Dilemma?? An Analytical and Prospective Study. Indian J Otolaryngol Head Neck Surg 2019;71:367–70. https://doi.org/10.1007/s12070-019-01603-3.

73. Mehle ME, Kremer PS. Sinus CT Scan Findings in "Sinus Headache" Migraineurs. Headache 2007;0:070516095856009-??? https://doi.org/10.1111/j.1526-4610.2007.00811.x.

74. Mohebbi A, Memari F, Mohebbi S. Endonasal Endoscopic Management of Contact Point Headache and Diagnostic Criteria. Headache: The Journal of Head and Face Pain 2010;50:242–8. https://doi.org/10.1111/j.1526-4610.2009.01520.x.

75. Mokbel KM, Abd Elfattah AM, Kamal E-S. Nasal mucosal contact points with facial pain and/or headache: lidocaine can predict the result of localized endoscopic resection. Eur Arch Otorhinolaryngol 2010;267:1569–72. https://doi.org/10.1007/s00405-010-1245-8.

76. Moretz WH, Kountakis SE. Subjective Headache before and after Endoscopic Sinus Surgery. American Journal of Rhinology 2006;20:305–7. https://doi.org/10.2500/ajr.2006.20.2856.

77. Parsons DS, Batra PS. Functional Endoscopic Sinus Surgical Outcomes for Contact Point Headaches. Laryngoscope 1998;108:696–702. https://doi.org/10.1097/00005537-199805000-00013.

78. Patel ZM, Kennedy DW, Setzen M, Poetker DM, DelGaudio JM. “Sinus headache”: rhinogenic headache or migraine? An evidence-based guide to diagnosis and treatment: “Sinus headache”: rhinogenic headache or migraine? INTERNATIONAL FORUM OF ALLERGY AND RHINOLOGY 2013;3:221–30. https://doi.org/10.1002/alr.21095.

79. Patel ZM, Setzen M, Poetker DM, DelGaudio JM. Evaluation and Management of “Sinus Headache” in the Otolaryngology Practice. Otolaryngologic Clinics of North America 2014;47:269–87. https://doi.org/10.1016/j.otc.2013.10.008.

80. Paulson EP, Graham SM. Neurologic Diagnosis and Treatment in Patients with Computed Tomography and Nasal Endoscopy Negative Facial Pain: The Laryngoscope 2004;114:1992–6. https://doi.org/10.1097/01.mlg.0000147935.59755.45.

81. Pedram D, Jennifer H, John M. “Sinus Headache” in Chronic Rhinosinusitis (CRS): An Analysis of Otolaryngology Initiated Treatment Efficacy of Migraine in the CRS Patient. Journal of Sinusitis and Migraine 2016;1:1–4.

82. Perry BF, Login IS, Kountakis SE. Nonrhinologic Headache in a Tertiary Rhinology Practice. Otolaryngology–Head and Neck Surgery 2004;130:449–52. https://doi.org/10.1016/j.otohns.2004.01.005.

83. Peter J C. Analysis of headache outcomes in patients treated with nasal and sinus surgery. Arch Otolaryngol Rhinold 2019:020–5. https://doi.org/10.17352/aor.000090.

84. Rai U, Devi P, Singh N, Lyngdoh N, Sudhiranjan T, Nongthombam N. Contact point headache: Diagnosis and management in a tertiary care center in Northeast India. J Med Soc 2018;32:51. https://doi.org/10.4103/jms.jms_69_16.

85. Ramadan HH. Nonsurgical versus Endoscopic Sinonasal Surgery for Rhinogenic Headache. American Journal of Rhinology 1999;13:455–7. https://doi.org/10.2500/105065899781329656.

86. Rebeiz EE, Rastani K. Sinonasal facial pain. Otolaryngologic Clinics of North America 2003;36:1119–26. https://doi.org/10.1016/S0030-6665(03)00122-1.

87. Roozbahany NA, Nasri S. Nasal and paranasal sinus anatomical variations in patients with rhinogenic contact point headache. Auris Nasus Larynx 2013;40:177–83. https://doi.org/10.1016/j.anl.2012.07.007.

88. Ryan RE, Kern EB. Rhinologic Causes of Facial Pain and Headache. Headache 1978;18:44–50. https://doi.org/10.1111/j.1526-4610.1978.hed1801044.x.

89. Sadeghi M, Saedi B, Ghaderi Y. Endoscopic Management of Contact Point Headache in Patients Resistant to Medical Treatment. Indian J Otolaryngol Head Neck Surg 2013;65:415–20. https://doi.org/10.1007/s12070-013-0629-7.

90. Sanderson RJ, Rivron RP. The effect of septal surgery on nasal symptoms. Rhinology 1992;30:17–20.

91. Sanges G, Feleppa M, Gamerra M, Sorrentino G, Luca R, Merone M, et al. Fronto-turbinalis Sinus Expansion and Headache. Curr Pain Headache Rep 2011;15:308–13. https://doi.org/10.1007/s11916-011-0194-2.

92. Schønsted-Madsen U, Stoksted P, Christensen P-H, Koch-henriksen N. Chronic headache related to nasal obstruction. J Laryngol Otol 1986;100:165–70. https://doi.org/10.1017/S0022215100098911.

93. Schreiber CP, Hutchinson S, Webster CJ, Ames M, Richardson MS, Powers C. Prevalence of Migraine in Patients With a History of Self-reported or Physician-Diagnosed "Sinus" Headache. Archives of Internal Medicine 2004;164:1769. https://doi.org/10.1001/archinte.164.16.1769.

94. Şenbil, N.; Gürer, Y.K.Y.; Üner, Ç.; Barut, Y. Sinusitis in children and adolescents with chronic or recurrent headache: a case–control study. J. Headache Pain 2008, 9, 33–36, doi:10.1007/s10194-008-0007-0.

95. Sindwani R, Wright ED. Role of endoscopic septoplasty in the treatment of atypical facial pain. J Otolaryngol 2003;32:77–80.

96. Smith BC, George LC, Svider PF, Nebor I, Folbe AJ, Sheyn A, et al. Rhinogenic headache in pediatric and adolescent patients: an evidence-based review. Int Forum Allergy Rhinol 2019;9:443–51. https://doi.org/10.1002/alr.22268.

97. Sollini G, Mazzola F, Iandelli A, Carobbio A, Barbieri A, Mora R, et al. Sino-Nasal Anatomical Variations in Rhinogenic Headache Pathogenesis: Journal of Craniofacial Surgery 2019;30:1503–5. https://doi.org/10.1097/SCS.0000000000005239.

98. Stammberger H, Wolf G. Headaches and Sinus Disease: The Endoscopic Approach. Ann Otol Rhinol Laryngol 1988;97:3–23. https://doi.org/10.1177/00034894880970S501.

99. Swain SK, Behera IC, Mohanty S, Sahu MC. Rhinogenic contact point headache – Frequently missed clinical entity. Apollo Medicine 2016;13:169–73. https://doi.org/10.1016/j.apme.2016.08.001.

100. Tosun F, Gerek M, Ozkaptan Y. Nasal Surgery for Contact Point Headaches. Headache 2000;40:237–40. https://doi.org/10.1046/j.1526-4610.2000.00034.x.

101. Wang J, Yin J-S, Peng H. Investigation of diagnosis and surgical treatment of mucosal contact point headache. Ear Nose Throat J 2016;95:E39-44.

102. Wang J, Yin J, Peng H. Diagnosis and Surgical Treatment of Mucosal Contact Point Headache: Mucosal Contact Point Headache May Not be Accurately Diagnosed before Surgery. SM Otolaryngology 2017;1:1003.

103. Wee JH, Lee J-E, Hong S-L, Shin JM, Kim D-Y. Prospective Study on the Characteristics and Postoperative Improvement of Rhinogenic Headache. J Rhinol 2015;22:6. https://doi.org/10.18787/jr.2015.22.1.6.

104. Welge-Luessen A, Hauser R, Schmid N, Kappos L, Probst R. Endonasal Surgery for Contact Point Headaches: A 10-Year Longitudinal Study: The Laryngoscope 2003;113:2151–6. https://doi.org/10.1097/00005537-200312000-00019.

105. West B, Jones NS. Endoscopy-Negative, Computed Tomography-Negative Facial Pain in a Nasal Clinic: The Laryngoscope 2001;111:581–6. https://doi.org/10.1097/00005537-200104000-00006.

106. Wilkie MD, Leong SC. Nonsinogenic facial pain and the role of the sino-nasal outcome test. Pain Management 2015;5:157–60. https://doi.org/10.2217/pmt.15.11.

107. Yarmohammadi ME, Ghasemi H, Pourfarzam S, Nadoushan MRJ, Majd SA. Effect of turbinoplasty in concha bullosa induced rhinogenic headache, a randomized clinical trial. J Res Med Sci 2012;17:229–34.

108. Yazici ZM, Cabalar M, Sayin İ, Kayhan FT, Gurer E, Yayla V. Rhinologic Evaluation in Patients With Primary Headache: Journal of Craniofacial Surgery 2010;21:1688–91. https://doi.org/10.1097/SCS.0b013e3181f3c666.
